# Supplementary material for: Cerebral small vessel disease burden is associated with decreased abundance of gut Barnesiella intestinihominis bacterium in the Framingham Heart Study
Source: Sci Rep. 2023 Aug 21;13:13622. doi: 10.1038/s41598-023-40872-5 (PMC10442369; doi:10.1038/s41598-023-40872-5)
Supplement: Supplementary file 1 — Supplementary Figures. [file 41598_2023_40872_MOESM1_ESM.docx]

***Cerebral Small Vessel Disease Burden is Associated with Decreased Abundance of Gut Barnesiella intestinihominis Bacterium in the Framingham Heart Study***

Bernard Fongang^1,2,3,#^, Claudia L. Satizabal^1,3,5,7^, Tiffany F. Kautz^1,4^, Yannick W. Ngouongo^1^, Jazmyn A. SherraeMuhammad^1^, Erin Vasquez^1^, Julia Mathews^1^, Monica Goss^1^, Amy R. Saklad^1^, Jayandra Himali^1,3,5,6,7^, Alexa Beiser^5,6,7^, Jose E. Cavazos^1,8^, Michael C. Mahaney^9^, Gladys Maestre^10^, Charles DeCarli^11^, Eric L. Shipp^1^, Ramachandran S. Vasan^5,12,13,14,15,16,17^, Sudha Seshadri^1,5,8,18^.

1. Glenn Biggs Institute for Alzheimer's & Neurodegenerative Diseases, University of Texas Health Science Center at San Antonio, San Antonio, Texas, United States of America.
2. Department of Biochemistry and Structural Biology, University of Texas Health Science Center at San Antonio, San Antonio, Texas, United States of America.
3. Department of Population Health Sciences, University of Texas Health Science Center at San Antonio, San Antonio, Texas, United States of America.
4. Department of Medicine, University of Texas Health Science Center at San Antonio, San Antonio, Texas, United States of America.
5. Framingham Heart Study, Framingham, Massachusetts, United States of America.
6. Department of Biostatistics, Boston University School of Public Health, Boston, Massachusetts, United States of America.
7. Department of Neurology, Boston University School of Medicine, Boston, Massachusetts, United States of America.
8. Department of Neurology, University of Texas Health Science Center at San Antonio, San Antonio, Texas, United States of America.
9. South Texas Diabetes and Obesity Institute and Department of Human Genetics, The University of Texas Rio Grande Valley School of Medicine, Brownsville, Texas, United States of America.
10. Department of Neurosciences and Department of Human Genetics, University of Texas Rio Grande Valley School of Medicine, Brownsville, Texas, United States of America.
11. Alzheimer's Disease Center, Department of Neurology, University of California, Davis, Sacramento, California, United States of America.
12. Department of Medicine, Section of Cardiovascular Medicine, Boston Medical Center, Boston University School of Medicine, Boston, Massachusetts, United States of America.
13. Department of Medicine, Section of Preventive Medicine and Epidemiology, Boston University School of Medicine, Boston, Massachusetts, United States of America.
14. Department of Epidemiology, Boston University School of Public Health, Boston, Massachusetts, United States of America.
15. Boston University's Center for Computing and Data Sciences, Boston, Massachusetts, United States of America.
16. The University of Texas School of Public Health in San Antonio, San Antonio, Texas, United States of America.
17. The Long School of Medicine, University of Texas Health Science Center, San Antonio, Texas, United States of America.
18. Department of Neurology, Boston University School of Medicine, Boston, Massachusetts, United States of America.

#: corresponding author: fongang@uthscsa.edu

**Key words**: Gut microbiome, Gut-microbiota-brain axis, Dementia, Alzheimer’s disease, small vessel disease, neuroimaging, neuropsychological test, 16S rRNA.

**Supplementary File SF1: Supplementary Figures**

**Figure S1: Study flowchart**

**Figure S2: cSVD markers and covariates distribution**

**Figure S3: Multivariate association analysis:** volcano plots of PSMD

**Figure S4: Multivariate association analysis:** volcano plots of WMH

**Figure S5: Multivariate association analysis:** volcano plots of EF

**Figure S6-8: Multivariate association analysis:** Scatter plots of PSMD depicting statistically significant associations at the phylum, genus, and species levels

**Figure S9-11: Multivariate association analysis:** Scatter plots of WMH depicting statistically significant associations at the phylum, genus, and species levels

**Figure S12-14: Multivariate association analysis:** Scatter plots of EF depicting statistically significant associations at the phylum, genus, and species levels

**Figure S15-17: Differential abundance analysis:** Differential abundance of the gut microbiome and measures of PSMD, WMH, EF

**Figure S18-20:**  **Alpha diversity measures** (ACE, Chao1, Observed, Shannon, and Simpson indexes) for EF, PSMD, WMH stratified by burden groups

**Figure S21: Correlation between measures of beta-diversity** (min_bray, min_wunifrac), alpha-diversity (ACE, Observed, Chao1, Simpson, and Shannon indexes), and cSVD markers after adjusting for age, age2, sex, BMI, and the time difference between the stool collection and MRI scans

**Figure S22:** **Principal Coordinate Analysis** depicting the diversity distribution between the different PSMD burden groups

**Figure S23-25:** **Predicted functional role of the microbial communities associated with PSMD, WMH, EF**

# 1. Study Flowchart


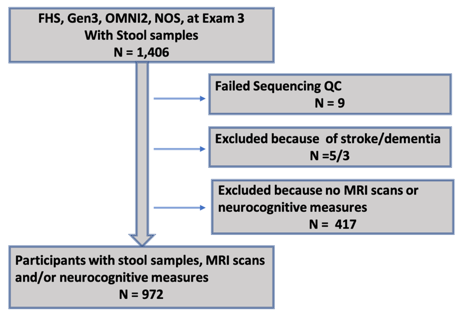


**Figure S1: Study flowchart**

# 2. cSVD markers and Covariates dependence


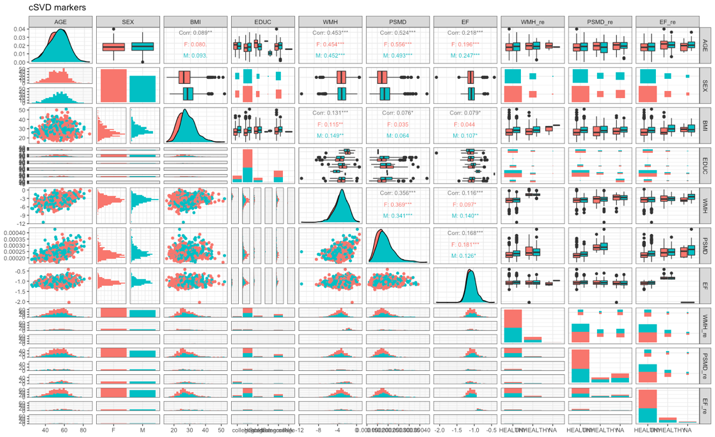


**Figure S2: cSVD markers and covariates distribution.** PSMD_re, WMH_re, and EF_re are stratified measures of PSMD, WMH, and EF , respectively as described in the text**.**

# 2. Multivariable association analysis

## 2.1 Volcano plots

### 2.1.1 PSMD volcano plots

| **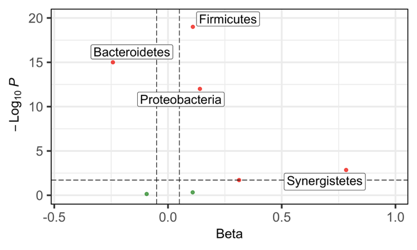** |
| --- |
| **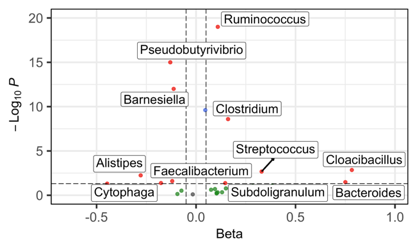** |
| **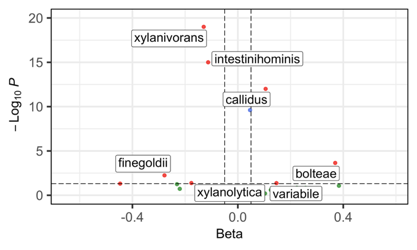** |
| **Figure S3: Multivariate association analysis:** volcano plots of PSMD depicting associations at the phylum, genus, and species levels (top to down) |

### 2.1.2 WMH volcano plots

| **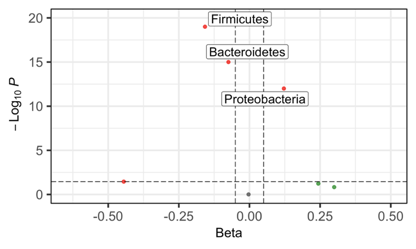** |
| --- |
| **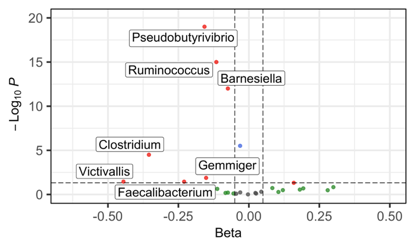** |
| **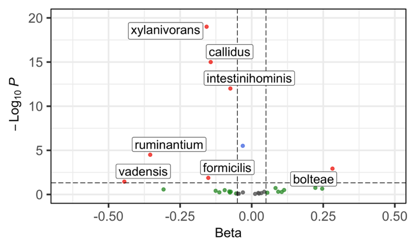** |
| **Figure S4: Multivariate association analysis:** volcano plots of WMH depicting associations at the phylum, genus, and species levels (top to down) |

### 2.1.3 EF volcano plots

| **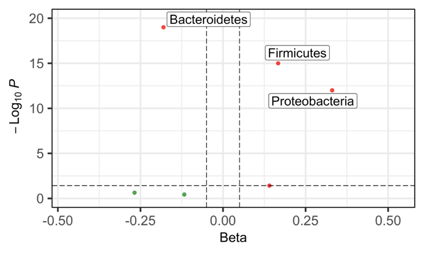** |
| --- |
| **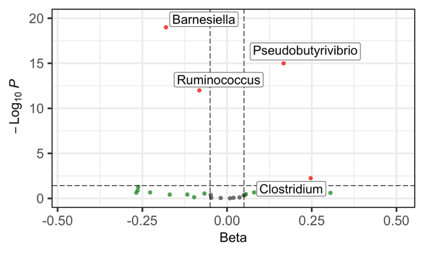** |
| **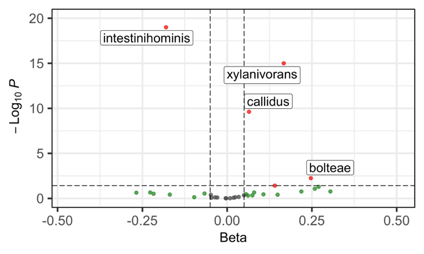** |
| **Figure S5: Multivariate association analysis:** volcano plots of EF depicting associations at the phylum, genus, and species levels (top to down) |

## 2.2 Scatter plots of significant taxa

### 2.2.1 Scatter plots of significant taxa associated with PSMD

**Figure S6: Multivariate association analysis:** Scatter plots of PSMD depicting statistically significant associations at the phylum level

**Figure S7: Multivariate association analysis:** Scatter plots of PSMD depicting statistically significant associations at the genus level

**Figure S8: Multivariate association analysis:** Scatter plots of PSMD depicting statistically significant associations at the species level

### 2.2.2 Scatter plots of significant taxa associated with WMH

**Figure S9: Multivariate association analysis:** Scatter plots of WMH depicting statistically significant associations at the phylum level

**Figure S10: Multivariate association analysis:** Scatter plots of WMH depicting statistically significant associations at the genus level

**Figure S11: Multivariate association analysis:** Scatter plots of WMH depicting statistically significant associations at the species level

### 2.2.3 Scatter plots of significant taxa associated with EF

**Figure S12: Multivariate association analysis:** Scatter plots of EF depicting statistically significant associations at the phylum level

**Figure S13: Multivariate association analysis:** Scatter plots of EF depicting statistically significant associations at the genus level

**Figure S14: Multivariate association analysis:** Scatter plots of EF depicting statistically significant associations at the species level

# 3. Differential abundance analysis

## 3.1 Differentially abundant taxa, stratified by PSMD measures

| **Phylum** | 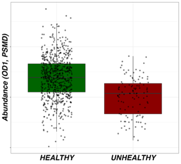 |  |  |
| --- | --- | --- | --- |
| **Genus** | 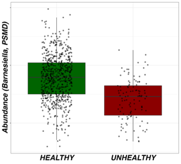 | 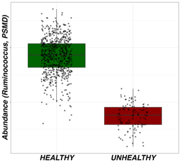 | 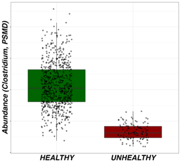 |
|  | 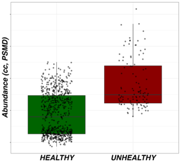 | 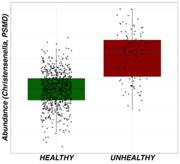 | 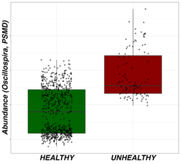 |
| **Species** | 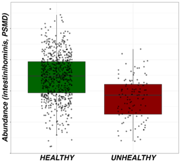 | 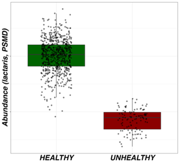 | 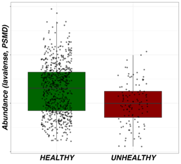 |
|  | 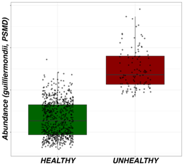 | 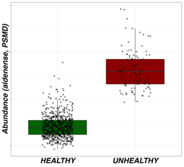 | 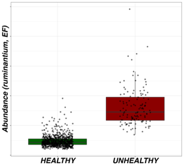 |

**Figure S15: Differential abundance analysis:** Differential abundance of the gut microbiome and measures of PSMD (Healthy = Lower burden, Unhealthy = High burden).

## 3.2 Differentially abundant taxa, stratified by WHM measures

| **Phylum** | 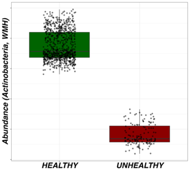 | 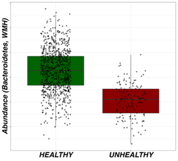 | 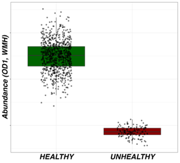 |
| --- | --- | --- | --- |
|  | 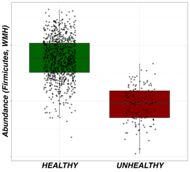 | 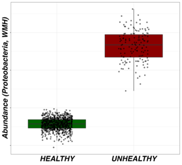 |  |
| **Genus** | 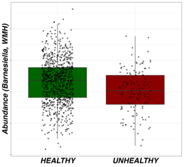 | 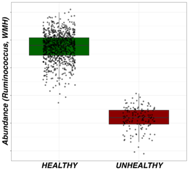 | 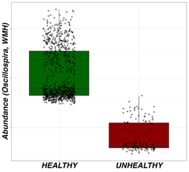 |
|  | 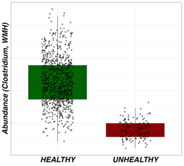 | 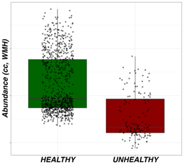 | 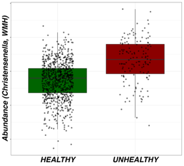 |
| **Species** | 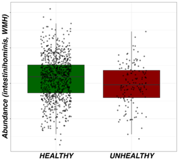 | 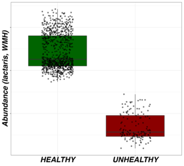 | 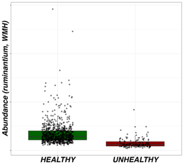 |
|  | 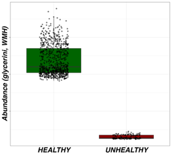 | 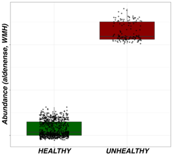 |  |

**Figure S16: Differential abundance analysis:** Differential abundance of the gut microbiome and measures of WMH (Healthy = Lower burden, Unhealthy = High burden).

## 3.3 Differentially abundant taxa, stratified by EF measures

| **Phylum** | 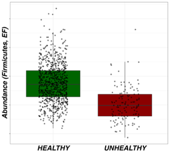 | 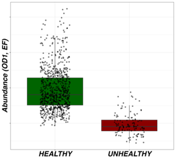 |  |
| --- | --- | --- | --- |
| **Genus** | 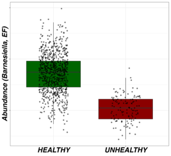 | 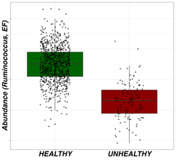 | 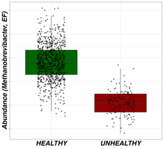 |
|  | 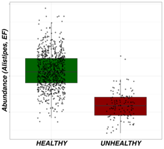 | 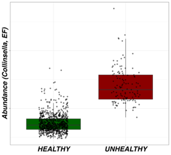 | 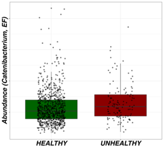 |
| **Species** | 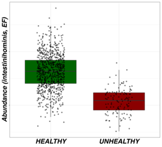 | 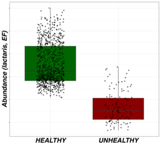 | 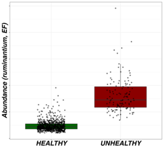 |
|  | 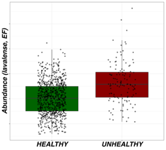 | 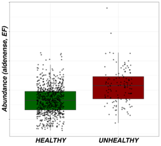 | 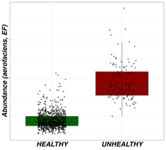 |

**Figure S17: Differential abundance analysis:** Differential abundance of the gut microbiome and measures of EF (Healthy = Lower burden, Unhealthy = High burden).

# 4. Diversity association analysis

## 4.1 Alpha diversity


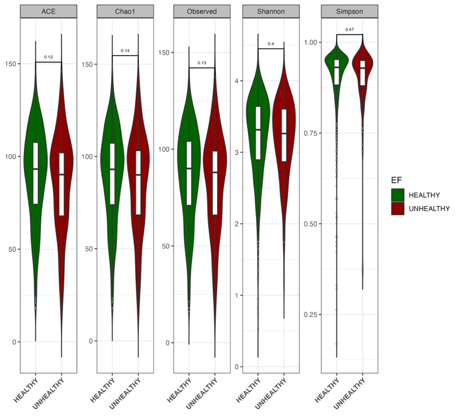


**Figure S18:**  **Alpha diversity measures (ACE, Chao1, Observed, Shannon, and Simpson indexes) for EF stratified by burden groups.** No statistically significant differences were found for all measures, indicating a relatively stable gut microflora composition amongst the two groups. significantly different between groups (Healthy = Lower burden, Unhealthy = High burden).


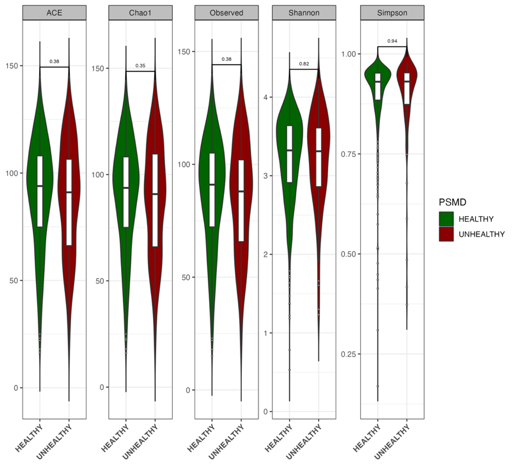


**Figure S19:**  **Alpha diversity measures (ACE, Chao1, Observed, Shannon, and Simpson indexes) for PSMD stratified by burden groups.** No statistically significant differences were found for all measures, indicating a relatively stable gut microflora composition amongst the two groups (Healthy = Lower burden, Unhealthy = High burden).


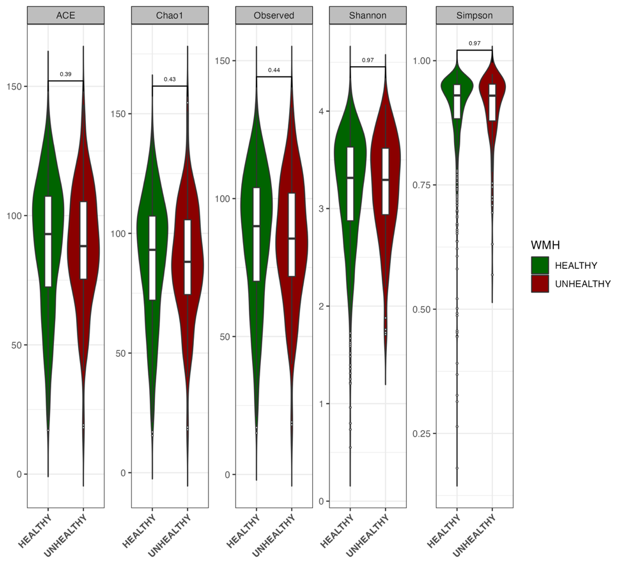


**Figure S20:**  **Alpha diversity measures (ACE, Chao1, Observed, Shannon, and Simpson indexes) for WMH stratified by burden groups.** No statistically significant differences were found for all measures, indicating a relatively stable gut microflora composition amongst the two groups (Healthy = Lower burden, Unhealthy = High burden).


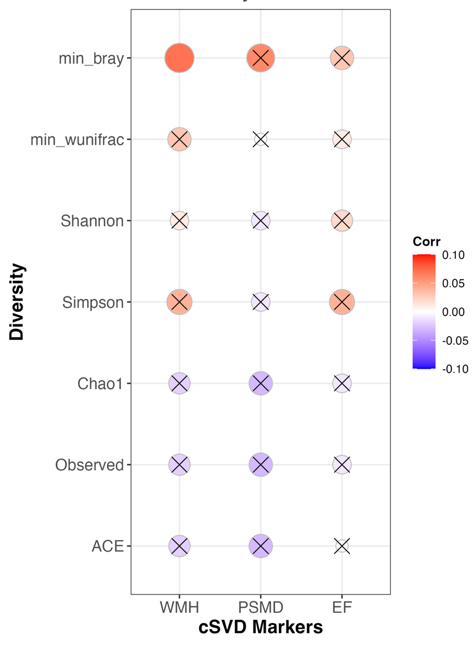


**Figure S21: Correlation between measures of beta-diversity (min_bray, min_wunifrac), alpha-diversity (ACE, Observed, Chao1, Simpson, and Shannon indexes) and cSVD markers after adjusting for age, age2, sex, BMI, and time difference between the stool collection and MRI scans**. No statistically significant correlations were found except for the association between WMH and minimum Bray-Curtis (min_bray). Just as in the stratified analysis, measures of alpha diversity did not associate with markers of cSVD. Crosses in the plot indicate that a correlation was not significant (unadjusted p-value >0.05); min_bray and min_wunfrac are defined in the text.

## 4.2 Beta diversity


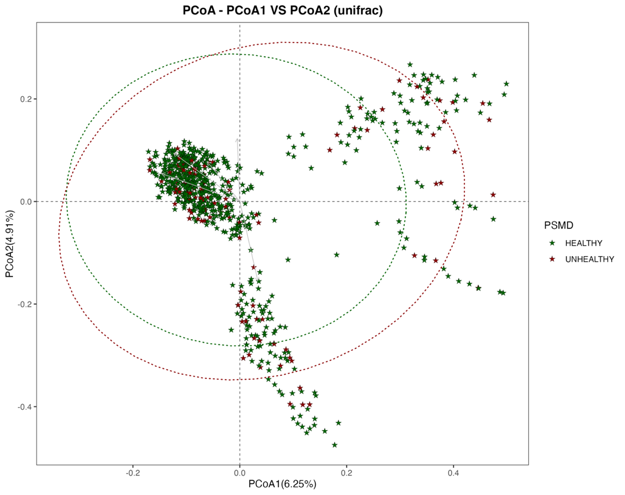


**Figure S22:** **Principal Coordinate Analysis (PCoA) depicting the diversity distribution between the different PSMD burden group**s. The different groups exhibit similar distribution. PCoA plot of unifrac distances for samples between WMH burden groups in microbial analysis (Healthy = Lower burden, Unhealthy = High burden).

# 5. Functional analysis with PICRUSt

| 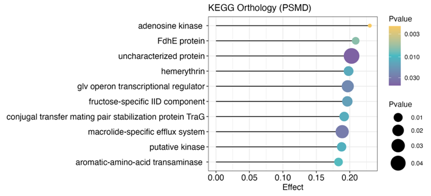 |
| --- |
| 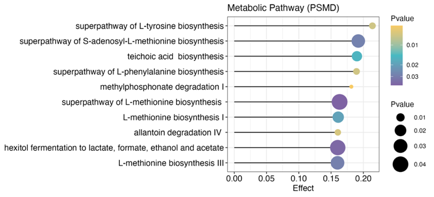 |
| 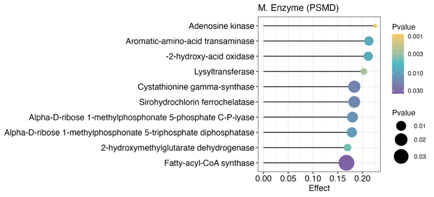 |

**Figure S23:** **Predicted functional role of the microbial communities associated with PSMD**

| 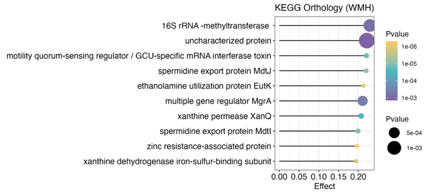 |
| --- |
| 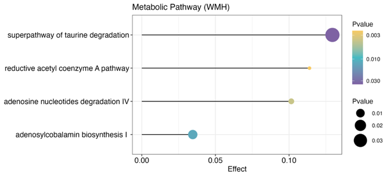 |
| 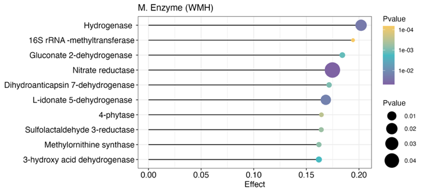 |

**Figure S24:** **Predicted functional role of the microbial communities associated with WMH**

| 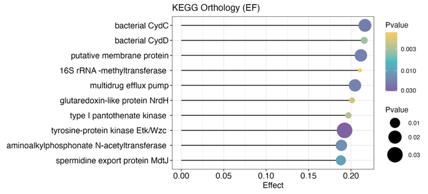 |
| --- |
| 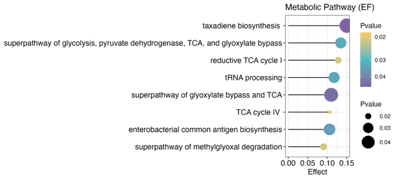 |
| 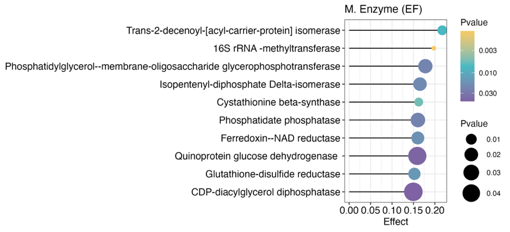 |

**Figure S25:** **Predicted functional role of the microbial communities associated with EF**
